# Contingency rules for pathogen competition and antagonism in a genetically based, plant defense hierarchy

**DOI:** 10.1002/ece3.5253

**Published:** 2019-05-23

**Authors:** Posy E. Busby, Gregory Crutsinger, Matthew Barbour, George Newcombe

**Affiliations:** ^1^ Botany and Plant Pathology Department Oregon State University Corvallis Oregon; ^2^ Department of Zoology University of British Columbia Vancouver British Columbia; ^3^ College of Natural Resources University of Idaho Moscow Idaho

**Keywords:** Cladosporium, eriophyid mite, fungal leaf endophyte, genetic resistance, *Melampsora*, microbiome, plant disease, plant–pathogen interaction, *Populus trichocarpa*, Trichoderma

## Abstract

1. Plant defense against pathogens includes a range of mechanisms, including, but not limited to, genetic resistance, pathogen‐antagonizing endophytes, and pathogen competitors. The relative importance of each mechanism can be expressed in a hierarchical view of defense. Several recent studies have shown that pathogen antagonism is inconsistently expressed within the plant defense hierarchy. Our hypothesis is that the hierarchy is governed by contingency rules that determine when and where antagonists reduce plant disease severity.
2. Here, we investigated whether pathogen competition influences pathogen antagonism using *Populus* as a model system. In three independent field experiments, we asked whether competition for leaf mesophyll cells between a *Melampsora* rust pathogen and a microscopic, eriophyid mite affects rust pathogen antagonism by fungal leaf endophytes. The rust pathogen has an annual, phenological disadvantage in competition with the mite because the rust pathogen must infect its secondary host in spring before infecting *Populus*. We varied mite–rust competition by utilizing *Populus* genotypes characterized by differential genetic resistance to the two organisms. We inoculated plants with endophytes and allowed mites and rust to infect plants naturally.
3. Two contingency rules emerged from the three field experiments: (a) Pathogen antagonism by endophytes can be preempted by host genes for resistance that suppress pathogen development, and (b) pathogen antagonism by endophytes can secondarily be preempted by competitive exclusion of the rust by the mite.
4. *Synthesis*: Our results point to a *Populus* defense hierarchy with resistance genes on top, followed by pathogen competition, and finally pathogen antagonism by endophytes. We expect these rules will help to explain the variation in pathogen antagonism that is currently attributed to context dependency.

## 1 INTRODUCTION

Plant microbiomes contain a diverse assemblage of fungi, bacteria, archaea, viruses, and even microscopic animals. These microorganisms can contribute to host defense (Zamioudis & Pieterse, 2012), but their contributions are typically contingent upon the host, pathogen, and the abiotic and biotic environment (reviewed by Busby, Ridout, & Newcombe, 2016). For example, one obvious contingency is that pathogen antagonism by fungal leaf endophytes should only occur if plants are genetically susceptible to the pathogen. Even this simple contingency creates a two‐level “defense hierarchy” in which genes for pathogen resistance preclude expression of pathogen antagonism. However, many more contingencies are likely within complex defense hierarchies that involve multiple pathogens and defense mechanisms. Without an understanding of the contingency rules that structure the hierarchy, pathogen antagonism can only be inconsistently applied to the benefit of agricultural practices (Busby et al., 2017; Dangl, Horvath, & Staskawicz, 2013; Ledford, 2015).

Plants typically host multiple pests and pathogens along with endophytes in their microbiomes. To the extent that pests and pathogens compete for the same host resource, depletion of that resource should limit the relative abundance of any one pathogen and therefore the degree of pathogen antagonism by endophytes. However, while the role of competition for structuring plant and animal communities has long been recognized (Callaway & Walker, 1997; Darwin, 1859; Diamond, 1978; Tilman, 1994), the ecological consequences of competition among microorganisms has attracted less attention (but see reviews Bever et al., 2010; Seabloom et al., 2015; Tollenarere, Susi, & Laine, 2016). This is so, despite the fact that most plants host multiple pathogens and despite the importance of disease as a primary structuring agent of plant communities.

The majority of studies evaluating the consequences of infection by multiple plant parasites have focused on competition among strains within the same species (Zhan & McDonald, 2013). When considering multiple pathogen species, arriving into the plant first (i.e., priority effect) can provide one pathogen with a competitive advantage over a later arriving pathogen (Al‐Naimi, Garrett, & Bockus, 2005). Expanding the scope of study to the broader microbial community, the immigration history of microbial symbionts (e.g., endophytes, mycorrhizae) can also influence plant disease outcomes (Adame‐Alvarez et al., 2014; Halliday, Umbanhowar, & Mitchell, 2017; Rua et al., 2013). However, the role of pathogen competition in the conditionality, or context dependency, of pathogen antagonism by endophytes is poorly understood.

**Figure 1:**
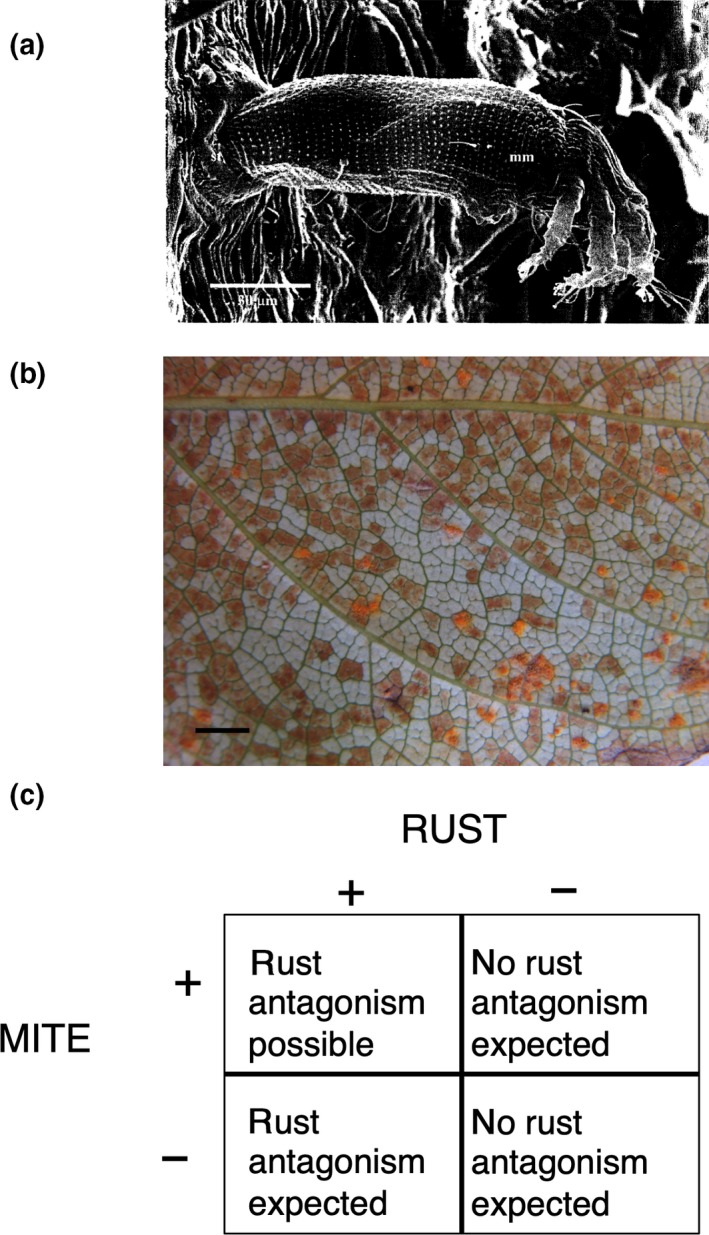
Scanning electron microscope image of an eriophyid mite (*S. mesophyllincola*) exiting a *P. trichocarpa* stomata (reprinted with permission from Hunt, 1992). Scale bar is 30 µm. (a) Mite bronzing on the underside of a *P. trichocarpa* leaf. Scale bar is 1 cm (b). Mite/rust combinations possible with major gene resistance to the two organisms and expected rust pathogen antagonism by fungal leaf endophytes

In this study, we evaluated whether competitive interactions in the microbiome reduce the extent of pathogen antagonism using *Populus trichocarpa* as a model system. Genes for resistance to pests and pathogens are well known in *Populus*. Pathogen antagonism is also well established: A diverse group of commonly occurring fungal leaf endophytes can antagonize *Melampsora* leaf rust in *Populus*, thereby reducing rust disease severity (Busby, Peay, & Newcombe, 2016; Raghavendra & Newcombe, 2013). However, a commonly occurring competitive interaction between the rust pathogen and a microscopic eriophyid mite, *Schizoempodium mesophyllincola* (Hunt, 1992; Oldfield, Hunt, & Gispert, 1998) (Figure 1a,b), could interfere with pathogen antagonism by endophytes.

Mite–rust pathogen competition includes a phenological factor of considerable importance. The mite migrates into the leaves of *P. trichocarpa* in early spring via stomata (Figure 1a) and begins consuming spongy mesophyll cells (Hunt, 1992). In competition for mesophyll cells, the mite thus has a head start on the rust pathogen which must first infect flushing needles of its aecial host, *Pseudotsuga menziesii*, before infecting *P. trichocarpa*, its telial host, later in the summer (Figure 1b) (Newcombe, Stirling, McDonald, & Bradshaw, 2000). Since rust fungi must parasitize living, undamaged mesophyll cells, mite‐damaged mesophyll will not support rust. Therefore, for both phenological and mechanistic reasons, we hypothesized that mites could competitively exclude the rust pathogen and thereby preempt rust pathogen antagonism by endophytes.

We tested the hypothesis that mite–rust competition preempts rust pathogen antagonism by endophytes in three independent field inoculation experiments. A known genetic basis for host resistance to both the mite and the rust pathogen allowed us to manipulate their presence in leaves by utilizing plant genotypes that varied in mite and rust resistance in manipulative experiments.

## 2 MATERIAL AND METHODS

### 2.1 Field inoculation experiments

Genetic resistance to *Melampsora* rust is well documented in *P. trichocarpa* and its hybrids (Newcombe, 1998; Newcombe, Bradshaw, & Chastagner, 1996); both major genes and quantitative genetic resistance contribute to defense against the pathogen. Major gene resistance, inherited not from *P. trichocarpa* but from the other *Populus* parent (e.g., *P. deltoides*, *P. nigra*), is “complete” in that plant recognition of the pathogen prevents its infection; quantitative resistance is “partial” in that many plant genes work together to limit damage once the pathogen has infected. Both complete and partial resistance to the mite have also been observed in *P. trichocarpa* and its hybrids (Newcombe, Muchero, & Busby, 2018). Importantly, while *P. trichocarpa* generally lacks complete resistance to both the mite and to rust, various hybrids can exhibit complete, major gene resistance to both the mite (Newcombe et al., 2018) and to rust (Newcombe, Bradshaw, et al., 1996). Therefore, we included genotypes of both *P. trichocarpa* and various hybrids in our experiments (31 genotypes total) to generate variation in major gene resistance to the mite and to rust. In previous, controlled greenhouse experiments (no mites) endophytes isolated from *P. trichocarpa* antagonized *Melampsora* rust equally in *P. trichocarpa* and hybrid genotypes (Raghavendra & Newcombe, 2013). Therefore, pathogen antagonism in the field was not expected to depend on the *Populus* hybrid per se, but rather on the degree to which mites and rust are able to infect the particular host. We expected the degree of antagonism by endophytes to occur in the following order for mite/rust combinations that are possible with major gene resistance to the two organisms (“−” resistance; “+” susceptibility) (Figure 1c):

-Mite+Rust>+Mite+Rust>-Mite-Rust=+Mite-Rust

Genetic variation in resistance to the mite and to the rust among our experimental *Populus* genotypes was not known a priori*;* rather it was inferred from the presence of mite bronzing (a discoloration of the underside of the leaf resulting from mite‐damaged plant cells, Figure 1b) and/or rust disease at the end of each experiment. If mites were capable of completely excluding rust, our inference of major gene resistance to the rust pathogen could be compromised. However, we very rarely observe leaves in the wild that are completely bronzed. The environment, rather than resistance genes could also preclude mites or rust from infecting plants and thus invalidate our inferences of major gene resistance. However, this is also unlikely because we observed both mite bronzing and rust disease on the leaves of wild *P. trichocarpa* growing adjacent to each field experiment, indicating that the organisms were present at each of the three field sites. Moreover, our inferences should be robust to potential environmental effects on mite and rust resistance given that we conducted common garden experiments in three different locations, each separated by approximately 600 km.

In each of the three field experiments, we inoculated plants with endophytes using standard protocols (Raghavendra & Newcombe 2013), allowed mites and rust to infect plants naturally, and collected data on rust and mite severity at the end of the growing season. Each experiment is described in detail below, and summarized in Table 1. Experiment 1 was conducted in Jefferson, Oregon, and included *Populus* hybrid genotypes only; experiment 2 was conducted in Mt. Vernon, Washington, and included both *Populus* hybrid and *P. trichocarpa* genotypes, and experiment 3 was conducted in Vancouver, British Columbia (BC), and included *P. trichocarpa* genotypes only.

**Table 1:** Details for each of the three field inoculation experiments testing rust pathogen antagonism by fungal leaf endophytes

	Location	Plant material	Endophyte treatments	Inoculation protocol
Exp1	Jefferson, Oregon	*Populus* hybrids (−M + R or −M−R)	Pathogen antagonists *Stachybotrys* sp. and *Trichoderma atroviride*	Endophytes inoculated with arrival of rust pathogen
Exp2	Mt. Vernon, Washington	*P. trichocarpa* (+M + R) and hybrids (−M + R or −M−R)	Pathogen antagonists *Trichoderma gamsii* and *Cladosporium tenuissimum*, pathogen facilitator (positive control) *Epicoccum nigrum*	Endophytes inoculated with arrival of rust pathogen
Exp3	Vancouver, British Columbia	*P. trichocarpa* (+M + R)	Pathogen antagonist *Cladosporium tenuissimum*, pathogen facilitator (positive control) *Epicoccum nigrum*, simplified endophyte community	Endophytes inoculated throughout the growing season, before and after arrival of rust pathogen

### 2.2 Experiment 1: *Populus* hybrids

We tested two rust pathogen antagonists, *Stachybotrys* sp. and *Trichoderma atroviride*, from a previous study (Raghavendra & Newcombe, 2013), against natural infection by *Melampsora* rust in a field inoculation experiment in Jefferson, OR (summer 2012). We used 20 *Populus* hybrid genotypes (including *P. trichocarpa* × *P. deltoides*, *P. trichocarpa* × *P. nigra*, *P. deltoides* × *P. trichocarpa*, and *P. deltoides* × *P. nigra*), with three tree replicates per genotype, treatment combination. Field‐planted cuttings were approximately six months old at the time of the experiment.

Leaves were inoculated either with an individual endophyte species or with sterile water in August, at the onset of natural infection by *Melampsora* rust. Cultures of *Stachybotrys* sp. and *T. atroviride* ranging from one‐to‐three weeks old were used to create inoculum. Spore concentration for each inoculum was approximately 7 × 10^5^ ml^−1^. Plants were sprayed with inoculum (or sterile water) at dusk and covered in plastic to maintain leaf moisture; bags were removed at dawn. Inoculated leaves (leaves at LPI positions 4–7) were flagged for later sampling.

In October, at the end of the growing season, we collected inoculated leaves and transported them to the laboratory for image analysis using Assess™ software. *Melampsora* severity was calculated as the percentage of leaf area covered by uredinia, the rust‐colored, asexual spores of *Melampsora* (Figure 1b). We observed no evidence of mite bronzing in any of the hybrid genotypes, so the severity of mite damage was not scored.

We used linear mixed‐effects models to test endophyte effects on rust severity (no transformation was needed to meet assumptions of normality). In these models, the leaf was used as the experimental replicate since endophytes infect leaves locally (Stone, Bacon, & White, 2000) and affect disease severity locally (Arnold et al., 2003; Raghavendra & Newcombe, 2013). Our model included plant genotype, endophyte treatment, and their interaction as fixed effects, and tree replicate as a random effect (nested within genotype) to account for local environmental effects on rust severity within the common garden. Mite damage was not included in models since mite bronzing did not occur. *p*‐Values for fixed effects were calculated from *F* tests based on Sattethwaite's approximation and for random effects are calculated based on likelihood ratio tests. We calculated both marginal and conditional *R* ^2^ values describing the proportion of variance explained by fixed factors, and both fixed and random factors, respectively (Nakagawa & Schielzeth, 2013). Finally, we used directional, planned contrasts to evaluate differences between each endophyte treatment group and the control group. For these analyses (and those described below), we used the *lmer* and *glht* function in the *lme4*, *lmerTEST*, and *multcomp* packages in R version 3.0.2 (R Core Team, 2015).

### 2.3 Experiment 2: *Populus trichocarpa* and hybrids

We tested two rust pathogen antagonists, *Trichoderma gamsii* and *Cladosporium tenuissimum*, from a more recent study (Busby, Peay, et al., 2016), against natural infection by *Melampsora* in a field inoculation experiment in Mt. Vernon, WA (summer 2014). We also included a negative control (sterile water), a positive control (*Epicoccum nigrum*, an endophytic pathogen facilitator that increases *Melampsora* severity in *P. trichocarpa*, Busby, Peay, et al., 2016), and a community treatment that included all three endophytes. The experiment included six tree genotypes: three *P. trichocarpa* and three hybrid *Populus* genotypes (*P. trichocarpa* × *P. deltoides*). Each genotype, treatment combination included three tree replicates. Field‐planted cuttings were approximately one‐year‐old at the time of the experiment.

We inoculated tagged leaves (leaf positions 4–6) with endophytes or sterile water in September. The inoculum was prepared using the same methods described in Experiment 1. For individual endophyte treatments, the spore concentration was 1 × 10^6^ spores/ml; the community inoculum included all three endophytes in roughly equal proportion at the same concentration, 1 × 10^6^ spores/ml. Plants were inoculated at dusk and misted several times throughout the night to ensure continual leaf moisture. We sampled tagged leaves in October. Leaf bronzing and rust severity were scored on each leaf using a categorical scale: 0 = no damage, 1 = 1%–6%, 2 = 7%–12%, 3 = 13%–25%, 4 = 26%–50%, or 5 = >50% (Dirzo & Domínguez, 1995). Preliminary analysis of these data indicated that the endophyte treatment had a marginally significant effect on rust disease severity for hybrid genotypes, but not for *P. trichocarpa* genotypes. Therefore, for hybrid genotypes only we collected additional fine‐scale data on uredinial density on the leaf surface. We counted uredinia within a 1‐cm^2^ panel in the five most heavily infected areas on each leaf and then calculated a leaf‐level mean value for rust disease severity.

We used linear mixed‐effects models to test endophyte effects on rust disease severity (log(*x* + 1)‐transformed to meet assumptions of normality) in *P. trichocarpa* and its hybrids (two separate models). Again, the leaf was the experimental replicate in models, and we included the endophyte treatment, plant genotype, mite bronzing (log(*x* + 1)‐transformed), and two‐way interactions as fixed effects, and plant as a random effect (nested within plant genotype). All hybrid *Populus* genotypes exhibited complete resistance to the mite; therefore, mite bronzing was not included in the hybrid model. *p*‐Values for fixed effects were calculated from *F* tests based on Sattethwaite's approximation; *p*‐value for the random effect was calculated using a likelihood ratio test.

### 2.4 Experiment 3: *Populus trichocarpa*

We tested the rust pathogen antagonist *Cladosporium tenuissimum* (Busby, Ridout, et al., 2016) against natural infection by *Melampsora* in a field inoculation experiment on the campus of the University of British Columbia in Vancouver, BC (summer 2014). In addition to *C. tenuissimum*, we included a negative control (sterile water), a positive control (*E. nigrum*, a pathogen facilitator that increases *Melampsora* rust severity, Busby, Peay, et al., 2016), and a simplified endophyte community. In addition to *C. tenuissimum* and *E. nigrum*, the community inoculum included species of *Stachybotrys*, *Fusarium*, *Truncatella*, *Phomopsis*, *Alternaria*, *Chaetomium*, *Xylaria*, *Curvularia*, *Phoma*, and two unknown endophyte species. All fungi were originally isolated from surface‐sterilized leaves of *P. trichocarpa* (collected from wild trees growing along the nearby Skagit river), and none of the fungi are known pathogens of *P. trichocarpa* (Newcombe, 1996). These fungi are therefore endophytic in *P. trichocarpa* according to standard definitions of endophytism (Stone et al., 2000). The five genotypes of *P. trichocarpa* included in this experiment are part of a genome‐wide association population (for further methodological details see McKown et al., 2014). The genotypes were selected with the prerequisites that they were equally related and represented trait variation within southern BC localities (latitude range: 49–52°N). For detailed tree propagation methods, see Crutsinger et al., 2014. We included nine replicate trees per genotype, endophyte treatment combination.

Trees were 3‐years‐old at the time of this field experiment (approximately 2–3 m in height), in 25‐gallon pots filled with potting soil. We inoculated leaves with endophytes (or sterile water for control) four times in the growing season, monthly, beginning in May, by spraying with inoculum containing fungal spores suspended in sterile water. To generate inoculum, we scraped spores and mycelium from one‐ to three‐week‐old pure cultures of each isolate (grown on PDA plates). For the community inoculum, we pooled spores from 2–3 plates per isolate. For each of the four inoculations, the spore concentration was standardized across fungal treatments (May and June: 2 × 10^7^ spores/ml, July: 5 × 10^7^ spores/ml and August: 2.5 × 10^7^ spores/ml). At dusk, on evenings with elevated relative humidity to increase fungal infection opportunities, we inoculated tagged leaves by spraying each plant with approximately 100 ml of inoculum, or 100 ml of sterile water for controls.

At the end of the growing season, we collected nine inoculated leaves from each tree (leaf positions 4–6, tagged in May) on three haphazardly selected lower canopy branches. We used the same categorical method described above to score mite bronzing and rust disease severity independently on each leaf (Dirzo & Domínguez, 1995). Because trees in experiment 3 were larger and had more branches than those in experiments 1 and 2, we were able to sample more leaf replicates and to calculate an average tree‐level damage score for both mite bronzing and rust disease severity: ∑*n_i_* (*C_i_*)/*N*, where *n_i_* is the number of leaves in the *i*th category of damage, *C_i_* is the midpoint of each category (*C*_0_ = 0, *C*_1_ = 3.5, *C*_2_ = 9, *C*_3_ = 18.5, *C*_4_ = 37.5, *C*_5_ = 75%), and *N* is the total number of leaves sampled (Dirzo & Domínguez, 1995). Unlike experiments 1 and 2, the disease severity response variable for experiment 3 was at the tree‐level, and therefore, tree was not included as a random effect in models. All factors in the models (plant genotype, fungal treatment, and their interaction) were fixed, and their significance was tested using *F* tests with Type II SS. Next, we used analysis of variance to test if the endophyte treatment or mite bronzing (log(*x* + 1)‐transformed) influenced rust disease severity (log(*x* + 1)‐transformed). Our model also included plant genotype and interactions between genotype and endophyte treatment, and genotype and mite bronzing. Statistical significance was tested using *F* tests with Type II SS.

## 3 RESULTS

### 3.1 Experiment 1: Hybrid *Populus* genotypes

None of the 20 hybrid *Populus* genotypes exhibited mite bronzing, despite the presence of mite bronzing on neighboring wild *P. trichocarpa*, suggesting that all 20 genotypes expressed major gene resistance to the local population of *S. mesophyllincola*. Moreover, 17 of the 20 total genotypes exhibited no rust disease, despite the presence of rust on neighboring wild *P. trichocarpa*, suggesting that these 17 genotypes expressed major gene resistance to the local *Melampsora* rust pathotype. We included only the three rust‐susceptible genotypes (one *P. trichocarpa* × *P. deltoides* and two *P. trichocarpa* × *P. nigra*) in a linear mixed‐effects model testing endophyte effects on rust disease severity (no transformation was needed to meet assumptions of normality).

**Figure 2:**
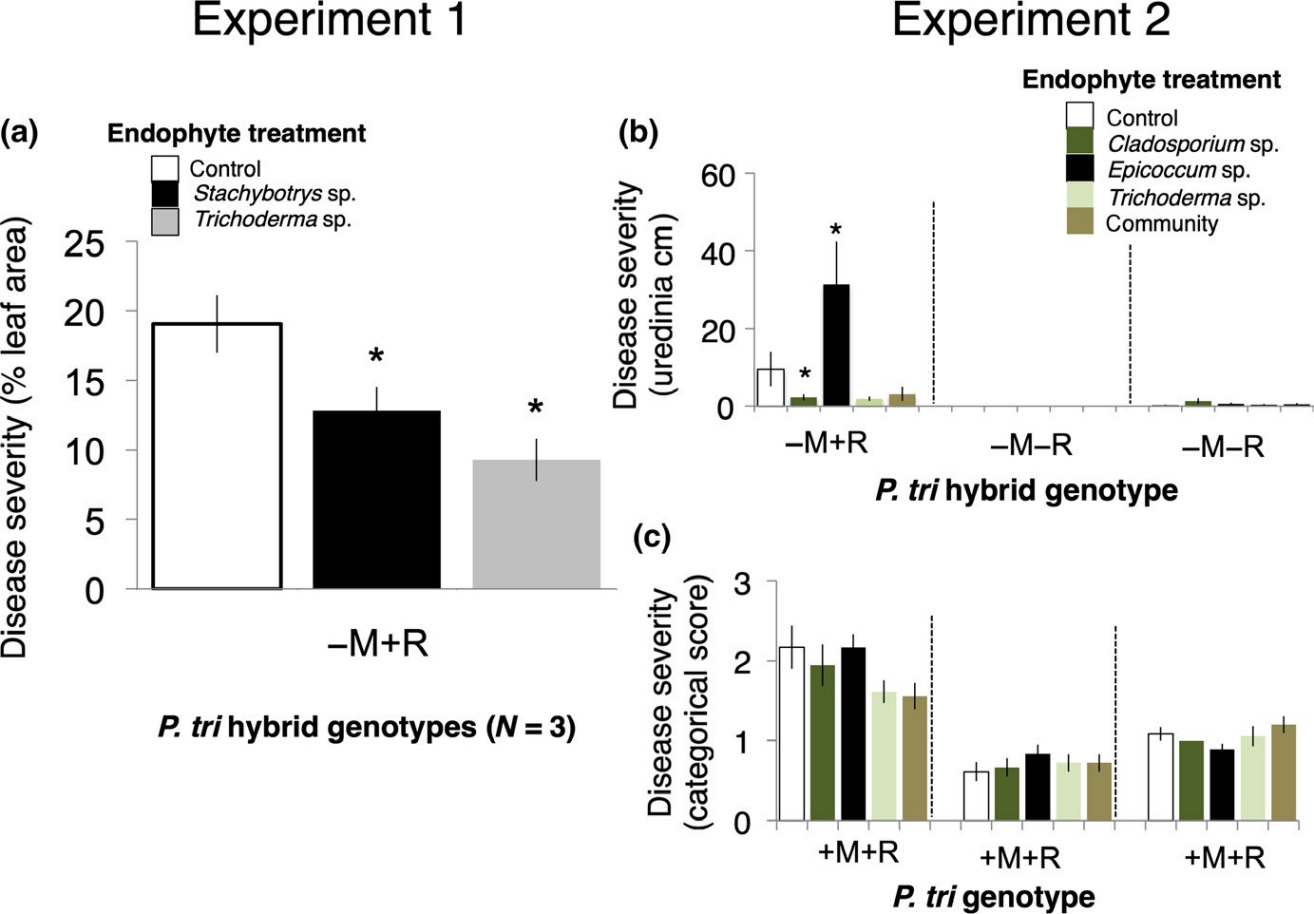
Endophytes antagonized the rust pathogen only when a major gene for rust resistance was absent but a major gene for mite resistance was present (* indicates treatment group mean differed significantly from control group mean, *p* < 0.05, error bars are *SE*). In experiment 1, endophytes antagonized rust in three *P. trichocarpa* hybrid genotypes susceptible to rust but resistant to the mite (a). In experiment 2, endophytes modified rust severity only in the *P. trichocarpa* hybrid genotype susceptible to rust but resistant to the mite, not in the *P. trichocarpa* hybrid genotypes resistant to both rust and mites (b); endophytes had no effect on rust disease severity for *P. trichocarpa* genotypes resistant to rust and susceptible to mites (c). In experiment 3, endophytes had no effect on rust disease severity for five *P. trichocarpa* genotypes with varying levels of susceptibility to the mite and to rust (negative result of experiment 3 not shown)

We found evidence of rust pathogen antagonism by endophytes in the three hybrid genotypes that were resistant to the mite, but susceptible to rust (i.e., −M + R). The endophyte treatment had a significant effect on rust disease severity (*F* = 3.6, *p* = 0.038; Table S1); tree genotype and genotype‐by‐endophyte interaction were nonsignificant. Univariate, directional planned contrast tests revealed significant differences between both the *Stachybotrys* treatment and the control group (*p* = 0.05, 33% reduction in disease severity) and between the *Trichoderma* treatment and control group (*p* = 0.005, 51% reduction in disease severity) (Figure 2a).

### 3.2 Experiment 2: *P. trichocarpa* and hybrid *Populus* genotypes

All *P. trichocarpa* genotypes exhibited mite bronzing and rust disease, consistent with the expectation that *P. trichocarpa* lacks major gene resistance to both *S. mesophyllincola* and *Melampsora* rust (+M + R). In contrast, two of the three hybrid *Populus* genotypes exhibited neither mite bronzing nor rust disease, suggesting major gene resistance to both the local *S. mesophyllincola* population and the local rust *Melampsora* strain (−M−R); the third hybrid genotype exhibited no bronzing but displayed rust disease, suggesting major gene resistance to the mite but not to the rust (−M + R).

In our *P. trichocarpa* model, tree genotype and tree replicate had significant effects on rust disease severity, though endophyte treatment, mite bronzing, and their interactions with tree genotype were nonsignificant (Table S2A). We found evidence of rust pathogen antagonism by endophytes only in the hybrid genotype resistant to the mite but susceptible to rust (i.e., −M + R; Figure 2b,c). In contrast to the *P. trichocarpa* model, the endophyte treatment‐by‐genotype effect had a significant effect on rust disease severity in the *Populus* hybrid model (Table S2B). Univariate, directional planned contrast tests revealed a significant difference between the control group and the positive control, the pathogen facilitator, *Epicoccum* (*p* = 0.002, 227% increase in disease severity). Mean rust disease severity was lower for the pathogen antagonist *Cladosporium* than for the control group, but this difference was not statistically significant (*p* = 0.055, 75% reduction in disease severity). *Trichoderma* and the fungal community treatment did not significantly impact rust disease severity.

Our model utilizing uredinial density data provided greater resolution on endophyte effects on rust disease severity in hybrid *Populus*. In this model, the endophyte treatment and its interaction with genotype were both statistically significant and planned contrast tests revealed the significance of both *Epicoccum* (*p* < 0.001) and *Cladosporium* (*p* = 0.037) as rust pathogen modifiers. *Trichoderma* (*p* = 0.073) and the endophyte community were not statistically significant (Table S2C; Figure 2b).

### 3.3 Experiment 3: *P. trichocarpa* genotypes

**Figure 3:**
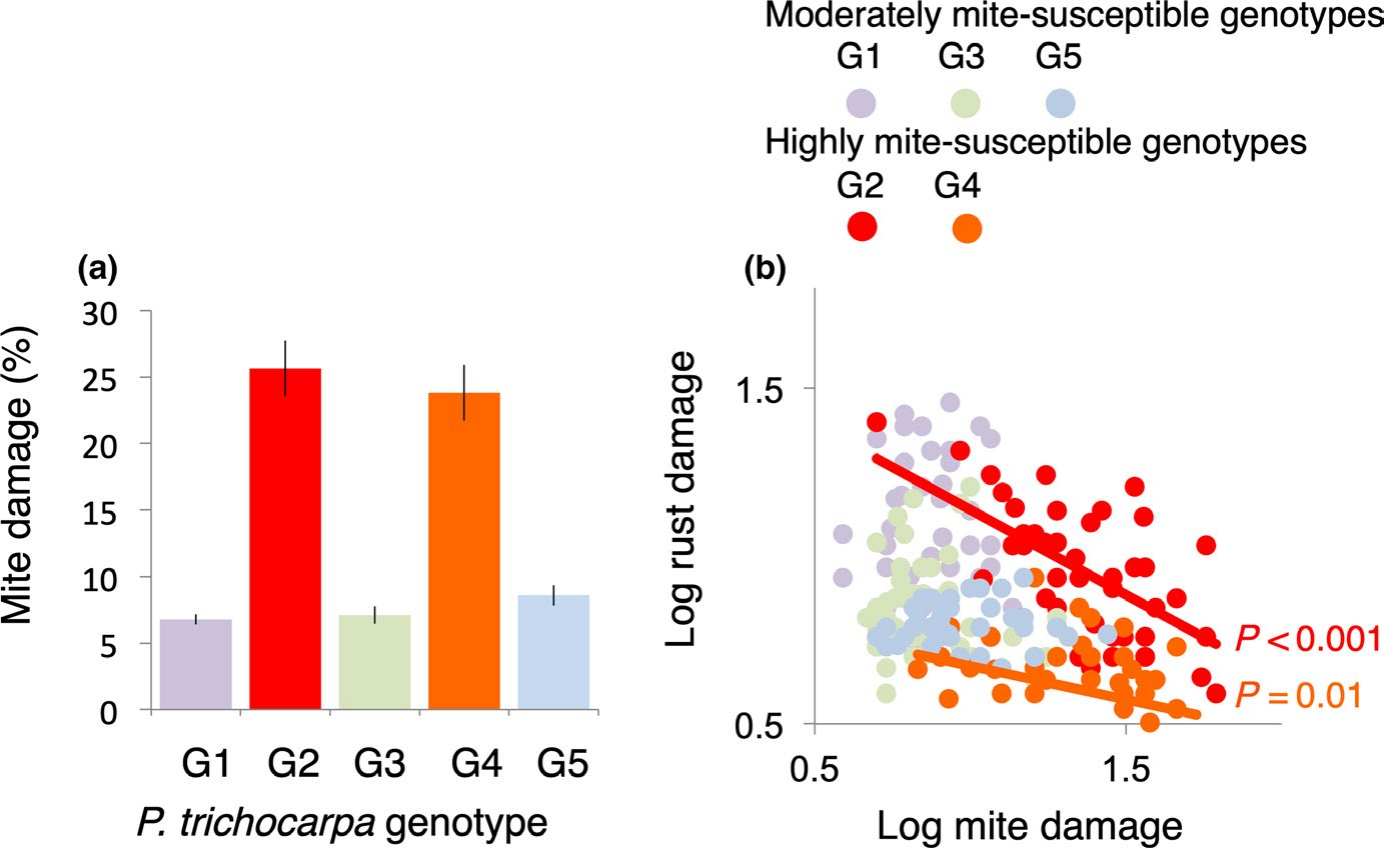
Mean bronzing severity (errors bars are *SE*) for five replicated genotypes of *P. trichocarpa* in the UBC common garden experiment (a). Heavily and moderately bronzed phenotypes differed significantly from each other. Bronzing severity was negative correlated with rust disease severity for heavily bronzed (i.e., highly mite‐susceptible) but not moderately bronzed (i.e., moderately mite‐susceptible) genotypes (b)

All five *P. trichocarpa* genotypes in the UBC common garden experiment exhibited both leaf bronzing and rust disease, indicating that each lacks major gene resistance to the local *S. mesophyllincola* population and to the local *Melampsora* rust strain (+M + R). And while leaf bronzing varied significantly among *P. trichocarpa* genotypes (Table S3; Figure 3a), neither the endophyte treatment nor endophyte‐by‐genotype factors influenced mite bronzing (Table S3). We observed two distinct bronzed phenotypes among the five tree genotypes: heavily bronzed and thus identified as highly susceptible to the mite (G2, G4) and lightly bronzed and thus identified as moderately susceptible to the mite (G1, G3, G5) (Figure 3a).

Rust disease severity also varied significantly among *P. trichocarpa* genotypes in the UBC garden (*F* = 45, *R* ^2 ^= 0.45, *p* < 0.001; Table S3), with mite bronzing and its interaction with plant genotype additionally contributing to disease severity (*F* = 10, *R* ^2 ^= 0.03, *p* = 0.002; *F* = 4.6, *R* ^2 ^= 0.05, *p* = 0.002; Table S3). Genotype‐specific models demonstrated that bronzing influenced rust disease severity for the highly mite‐susceptible plant genotypes (G2, G4) but not for the moderately susceptible genotypes (G1, G3, G5) (Table S3; Figure 3b). Neither the endophyte treatment nor its interaction with plant genotype influenced disease severity.

## 4 DISCUSSION

### 4.1 Contingency rules in a genetically based, plant defense hierarchy

Fungal endophytes can contribute to plant defense by antagonizing pathogens (Arnold et al., 2003; Busby, Peay, et al., 2016). But, pathogen antagonism is commonly reported to be context‐dependent (Busby, Ridout, et al., 2016), and the reasons for its dependency, or inconsistent expression, have not been clear. In our study, we tested whether pathogen antagonism is contingent upon both plant genetic resistance to a rust pathogen and also rust pathogen competition. In the latter case, we employed host genetic resistance to vary a competitive interaction between an endophytic mite and a rust pathogen for leaf mesophyll cells. We then asked how this competitive interaction affects rust pathogen antagonism by fungal leaf endophytes in three independent field inoculation experiments. Together, the results of our field experiments revealed two contingency rules. First, pathogen antagonism by endophytes can be preempted by host genes for resistance that suppress pathogen development. Second, antagonism can also be preempted by a competing pathogen. The first rule is a simple confirmation of the primacy of genetic resistance. However, the second rule is surprising because competition among unrelated pathogens, although explored in some contexts (Power, 1996) is rarely considered in the context of pathogen antagonism by endophytes. While rust antagonism by endophytes may occur even when mites strongly limit rust abundance, we were unable to detect such an effect in the field. We should note that our findings do not explain the context dependency of pathogen antagonism in controlled studies that commonly exclude all competing pathogens.

Our results illustrate a three‐level, defense hierarchy within the *Populus* microbiome dictated by two simple contingency rules. Major genes are at the top of the hierarchy since they can completely prevent infection by the rust pathogen. It thus makes sense, in light of our findings, for agricultural scientists to focus on single pathogens and host genetics since that is the top of our hierarchy. Pathogen competition is on the second level of the hierarchy since mites are also regulated by a major gene for resistance. Endophytic pathogen antagonists are on the third level since they can be regulated by either genes for resistance or second‐level mites. Because the contingency rules identified in our study are determined in part by the rust life cycle, we expect that the rules should apply to many other temperate‐zone plants that are telial hosts of heteroecious rust pathogens. These rusts, like *Melampsora* on *Populus*, are phenologically disadvantaged with respect to any competing pathogen, like the mite, that is synchronized with early growth of the host in the spring. In our study, the ability of mites to suppress pathogen antagonism by outcompeting rust likely depended on their early, spring arrival into the community (i.e., a priority effect), and the later arrival of the rust pathogen (experiments 1 and 2). However, even when we inoculated endophytes early in the growing season (experiment 3), we still saw no effect of endophytes on rust disease severity in the presence of mites. While we expect that immigration history influences whether antagonists or competitors more strongly limit pathogens, additional research on these poorly studied interactions is needed before general patterns can be discerned. Moreover, research is needed to determine whether plant resistance genes interact with the endophyte community in ways that impact disease severity. This may have occurred in our study, though we are unable to evaluate this possibility because we did not survey endophyte communities.

### 4.2 Community genetics

In our study, working with the model tree *Populus* allowed us to take advantage of ecologically relevant phenotypes and their underlying genetic architecture (Whitham et al., 2006). We found evidence that host genetics (i.e., resistance genes) play a primary role in determining rust–mite competitive interactions and the effects of competition on rust pathogen antagonism by endophytes. More specifically, a single *Populus* resistance gene determines not only resistance to the eriophyid mite *S. mesophyllincola* in TxD hybrids (Newcombe et al., 2018), but also whether pathogen competition or pathogen antagonism is more likely occur. By influencing rust disease severity, we therefore expect this gene to have cascading indirect effects on plant‐associated communities and ecosystem processes (e.g., leaf decomposition).

We also found evidence that intraspecific variation in mite resistance within *P. trichocarpa* can influence the degree to which rust is competitively excluded. In experiment 3, mite damage was negatively correlated with rust disease severity in *P. trichocarpa* genotypes highly susceptible to the mite, but not in *P. trichocarpa* genotypes expressing intermediate susceptibility. In other words, we observed a threshold effect whereby mites influenced rust disease severity only in tree genotypes where they were abundant. In this way, mites could alter the fitness landscape and selection for rust resistance. However, we found no evidence that variation in mite–rust competition within *P. trichocarpa* influenced antagonism of the rust pathogen by endophytes. Endophytes did not modify rust disease severity in the high or intermediate mite‐susceptible genotypes.

Geographic variation in species interactions is expected to influence when and where plant genes affect pathogen competition and antagonism. Our study focused on the western portion of the geographic range of *P. trichocarpa*, where the mite is widespread. In contrast, mites are not common in the portion of the tree's range that is east of the Cascades (G. Newcombe, pers. comm.). The *Populus* defense hierarchy on the west side of the Cascades may not apply to the east side of the Cascades where genetic resistance to *Melampsora* is also weaker (Dunlap & Stettler, 1996). Rust outbreaks east of the Cascades are also episodic rather than annual (G. Newcombe, pers. comm.), and that too should affect the defense hierarchy reported here. Thus, antagonism by endophytes may be more ecologically significant east of the Cascades where genes for rust resistance and mites are weaker or absent, respectively. Together, our results point to genetic mosaics of species interactions that may have paved the way for divergent strategies of plant defense (Thompson, 2005).

## CONFLICT OF INTEREST

None declared.

## AUTHOR CONTRIBUTIONS

PEB and GN conceived the project. PEB, MB, and GC conducted the field experiment at UBC. PEB conducted the field experiments in OR and WA, analyzed data, and wrote the manuscript. GN, MB, and GC contributed feedback on the manuscript.

## Data Availability

Data are available from the Dryad Digital Repository: https://doi.org/10.5061/dryad.3t5602f.
