# Supplementary material for: Contingency rules for pathogen competition and antagonism in a genetically based, plant defense hierarchy
Source: Ecol Evol. 2019 May 23;9(12):6860–8. doi: 10.1002/ece3.5253 (PMC6662256; doi:10.1002/ece3.5253)
Supplement: Supplementary file 1 [file ECE3-9-6860-s001.docx]

**Table S1.** Model results for experiment 1, including data for only the three rust-susceptible genotypes.

|  |  |  |  |  |
| --- | --- | --- | --- | --- |
|  | **df** | ***F*** | ***P*** | **pseudo-R^2^** |
| Endophyte | 2 | 3.5 | 0.038 |  |
| Genotype | 2 | 0.85 | 0.43 |  |
| Endophyte x Genotype | 4 | 0.08 | 0.99 | All fixed 0.1 |
|  | **df** | ***X*^2^** | ***P*** |  |
| Plant | 1 | 40 | <0.001 | 0.5 |
|  |  |  |  |  |
| **Univariate, directional planned contrasts** | | |  |  |
|  |  | **z-value** | ***P*** |  |
| control-*Stachybotrys* |  | 1.7 | 0.05 |  |
| control-*Trichoderma* |  | 2.6 | 0.0048 |  |

**Table S2.** Model results for experiment 2. Mite bronzing is not included in the hybrid *Populus* model because the genotypes exhibited complete resistance to the mite.

| **A. *P. trichocarpa model*** | **df** | ***F*** | ***P*** | **pseudo-R^2^** |
| --- | --- | --- | --- | --- |
| Genotype | 2 | 39 | <0.001 |  |
| Endophyte | 4 | 0.21 | 0.93 |  |
| Mite bronzing | 1 | 0.8 | 0.37 |  |
| Genotype x endophyte | 8 | 0.63 | 0.75 |  |
| Genotype x mite brozing | 2 | 2.1 | 0.37 | All fixed 0.4 |
|  | **df** | ***X*^2^** | ***P*** | **pseudo-R^2^** |
| Plant | 1 | 56 | <0.001 | 0.3 |

| **B. Hybrid *Populus* model, categorical scores** | **df** | ***F*** | ***P*** | **pseudo-R^2^** |
| --- | --- | --- | --- | --- |
| Genotype | 2 | 33 | <0.001 |  |
| Endophyte | 4 | 1.7 | 0.17 |  |
| Genotype x endophyte | 8 | 2.6 | 0.015 | All fixed=0.43 |
|  | **df** | ***X*^2^** | ***P*** | **pseudo-R^2^** |
| Plant | 1 | 81 | <0.001 | 0.33 |
|  |  |  |  |  |
| **Univariate, directional planned contrasts** | |  |  |  |
|  |  | **z-value** | ***P*** |  |
| control-*Epicoccum* |  | -2.9 | 0.002 |  |
| control-*Cladosporium* |  | 1.6 | 0.055 |  |
| control-*Trichoderma* |  | 0.012 | 0.49 |  |
| control-community |  | 1.4 | 0.17 |  |
|  |  |  |  |  |
| **C. Hybrid *Populus* model, urdeninial density** | **df** | ***F*** | ***P*** | **pseudo-R^2^** |
| Genotype | 2 | 34 | <0.001 |  |
| Endophyte | 4 | 3.05 | 0.022 |  |
| Genotype x endophyte | 8 | 3.3 | 0.003 | All fixed 0.44 |
|  | **df** | ***X*^2^** | ***P*** | **pseudo-R^2^** |
| Plant | 1 | 54 | <0.001 | 0.28 |
| **Univariate, directional planned contrasts** | |  |  |  |
|  |  | **z-value** | ***P*** |  |
| control-*Epicoccum* |  | -3.3 | <0.001 |  |
| control-*Cladosporium* |  | 1.8 | 0.037 |  |
| control-*Trichoderma* |  | 1.5 | 0.073 |  |
| control-community |  | 1.7 | 0.1 |  |

**Table S3.** Model results from experiment 3.

| **Mite model** | **Sum of Squares** | **R^2^** | **Df** | **F** | ***P*** |
| --- | --- | --- | --- | --- | --- |
| Plant genotype | 15612.7 | 0.46 | 4 | 48 | <0.001 |
| Endophyte | 486.6 | 0.014 | 3 | 2.0 | 0.1162 |
| Genotype x endophyte | 1560.5 | 0.046 | 12 | 1.6 | 0.09438 |
| Residuals | 15942.5 | 0.47 | 196 |  |  |

| **Full rust model** | **Sum of Squares** | **R^2^** | **Df** | **F** | ***P*** |
| --- | --- | --- | --- | --- | --- |
| Mite damage | 1.3787 | 0.025 | 1 | 10 | 0.0016 |
| Plant genotype | 24.4201 | 0.45 | 4 | 45 | <0.001 |
| Endophyte | 0.1083 | 0.002 | 3 | 0.27 | 0.85 |
| Mite x Genotype | 2.4667 | 0.045 | 4 | 4.6 | 0.0015 |
| Endophyte x Genotype | 0.3895 | 0.0072 | 12 | 0.24 | 1.0 |
| Residuals | 25.7567 |  | 191 |  |  |
|  | 54.52 |  |  |  |  |

| **Genotype rust models** |  |  |  |  |  |
| --- | --- | --- | --- | --- | --- |
| **G5: moderately mite-susceptible** | **Sum of Squares** | **R^2^** | **Df** | **F** | ***P*** |
| Mite damage | 1.2 | 0.079 | 1 | 3.2 | 0.08 |
| Residuals | 14 |  | 40 |  |  |
| **G1: moderately mite-susceptible** |  |  |  |  |  |
| Mite damage | 0.0204 | 0.0049 | 1 | 0.202 | 0.66 |
| Residuals | 4.1 |  | 41 |  |  |
| **G3: moderately mite-susceptible** |  |  |  |  |  |
| Mite damage | 0.57 | 0.081 | 1 | 3.7 | 0.061 |
| Residuals | 6.4 |  | 42 |  |  |
| **G2: highly mite-susceptible** |  |  |  |  |  |
| Mite damage | 4.6 | 0.37 | 1 | 25 | <0.001 |
| Residuals | 7.7 |  | 42 |  |  |
| **G4: highly mite- susceptible** |  |  |  |  |  |
| Mite damage | 1.4 | 0.097 | 1 | 4.5 | 0.041 |
| Residuals | 13 |  | 41 |  |  |
